# Life disturbance and hospital visit experiences among Chinese patients with benign prostatic hyperplasia: a qualitative study

**DOI:** 10.1186/s12875-024-02378-5

**Published:** 2024-05-03

**Authors:** ZiYan An, QiWei Zhou, JinPeng Shao, ShuWei Xiao, Kun Zhao, WenJie Wei, YangYang Wu, Yong Song, Qing Yuan, WeiJun Fu, Xu Zhang

**Affiliations:** 1 Department of Urology, Third Medical Center, PLA General Hospital, YongDing Road No. 69, Beijing, 100039 China; 2 Medical School of PLA, Beijing, 100853 China; 3 Department of Urology, Third Affiliated Hospital of Naval Military Medical University, Shanghai, 200438 China; 4 Department of Urology, Air Force Medical Center, Beijing, 100142 China

**Keywords:** Benign prostatic hyperplasia, Lower urinary tract symptoms, Quality of life, Qualitative interview

## Abstract

**Background:**

The impact of lower urinary tract symptoms (LUTS) on the quality of life of patients with benign prostatic hyperplasia (BPH) has been rarely reported. Additionally, the challenges faced by these patients in seeking medical care have often been overlooked. In order to explore the personal struggles caused by LUTS and the difficulties or barriers experienced by Chinese patients with BPH when seeking help, we conducted a qualitative interview study.

**Methods:**

Qualitative interviews were conducted among 46 patients with BPH who were hospitalized in three tertiary hospitals in China from July 2021 to November 2022. Grounded theory was adopted as the methodology for the qualitative study. After obtaining written informed consent from the study participants, semi-structured interviews were conducted according to the question guidelines. The interview process was audio-recorded; subsequently, the recordings were transcribed, coded, and thematically analyzed.

**Results:**

The difficulties faced by Chinese patients with BPH were classified into seven main themes: (i) disturbed life, (ii) mental burden, (iii) disease cognition and communication, (iv) delayed treatment, (v) medication status, (vi) hospital visits barriers, and (vii) medical insurance issues. Further, each theme was subdivided into 2–5 sub-themes.

**Conclusions:**

LUTS have a certain effect on the life and spirit of patients with BPH. These patients face different degrees of difficulties in treatment and hospital visits. Therefore, better healthcare systems and additional social support are crucial for improving the current plight of these patients.

**Supplementary Information:**

The online version contains supplementary material available at 10.1186/s12875-024-02378-5.

## Introduction

Benign prostatic hyperplasia (BPH) is a common disease in middle-aged and elderly men worldwide and is highly correlated with lower urinary tract symptoms (LUTS). Latest research in rural areas of Zhengzhou, Henan Province, China, indicates the overall prevalence of BPH is 10.04% [1]. The weighted overall prevalence of LUTS/BPH was 10.66% in China in male individuals aged over 50 years according to the 2011 data from China Health and Retirement Longitudinal Study [2]. Another study revealed that the overall prevalence of LUTS/BPH were 11.97% in China in male and the prevalence climbed up as 22.70% in those aged over 70 years [3]. Although the LUTS are not life-threatening, it severely affects the daily life and quality of life (QOL) of patients with BPH, and may cause psychological and emotional distress [4, 5]. Current studies have reported that patients with BPH have low consultation rates and fail to receive correct health guidance and timely treatment [5–8]. That is because most patients perceive LUTS as part of the normal aging process and believe that there is no need to seek medical help; on the other hand, other patients avoid seeking medical intervention as they are embarrassed about sexual or urinary problems [9].

In China, patients with BPH may also face challenges in seeking medical help and self-care owing to the traditional taboos on sexual or urinary problems, which are often overlooked and seldom discussed. Besides, China’s medical system covers hospitals at all levels, community health service centers, rural clinics, etc. Among them, hospitals are classified into tertiary, secondary and primary hospitals based on their size, level and function. Tertiary hospitals are generally comprehensive medical institutions with advanced medical equipment and professional medical teams, which can provide high-level medical services. Secondary hospitals provide comprehensive medical services within the region. Primary hospitals mainly provide basic health care services for community residents. The distribution of medical resources in China is inconsistent, with large urban hospitals having an abundance of high-quality medical resources compared to primary hospitals in towns. This discrepancy can make it difficult for patients with severe symptoms to receive the necessary medication or surgical treatment, particularly in regions with limited medical resources. As a result, many patients are forced to travel to large cities to seek better treatment, leading to a phenomenon known as “ecdemic medical care” [10]. These patients, referred to as ecdemic patients, may encounter additional difficulties in seeking medical care in unfamiliar surroundings, which can weaken their determination to treat the disease.

Understanding the personal experience of patients with BPH during treatment and discovering their actual difficulties and needs will help researchers improve the poor plight of these patients in existing medical settings. However, only a few qualitative studies explored life experiences and medical care-seeking behaviors of patients with BPH [10–14]. The information about these patients is insufficient in China. Therefore, in the present study, the qualitative research method was used to conduct semi-structured interviews among Chinese hospitalized patients with BPH to explore the effects of LUTS and investigate their needs and barriers during the treatment. We hope that the results of this study will offer valuable insights into the lived experiences of patients with BPH, draw attention to their needs, and inform efforts to optimize the medical system, strengthen health management, and improve the convenience of medical treatment.

## Methods

### Study design

Based on the purpose of our research, grounded theory was chosen as the theoretical framework. In this qualitative study, semi-structured interviews were conducted to explore the experience from the perspective of the patients and discover the social factors behind it. The interview guideline was discussed and developed by all authors. It consisted of 19 open questions and subsequent prompts designed to elicit participants’ knowledge, understanding and experiences of BPH/LUTS and hospital visit. This study was approved by the Ethics Committee of PLA General Hospital (No. S2021-498-01).

### Patient recruitment

Study participants were patients with BPH who were admitted to three tertiary hospitals between July 2021 and November 2022. Grounded theory usually starts with purposive sampling and select participants who can best contribute to the developing theory. We developed loose inclusion/exclusion criteria, and used purposive sampling methods to select people with different experiences under the same disease to explore multiple dimensions of the social phenomenon under study and collect rich information [11]. The study followed the principles of information saturation, and recruitment was discontinued when no more new themes were identified. The inclusion criteria were as follows: (i) BPH patients aging from 50 to 85 years; (ii) with moderate to severe LUTS; (iii) willing to share the medical treatment experience of BPH; (iv) agreed to the recording of the interview and signed a written informed consent form. The exclusion criteria were as follows: (i) BPH patients with speech disorder or mental illness; (ii) unable to communicate normally without assistance.

### Setting

The First Medical Center and the Third Medical Center of the PLA General Hospital are both large tertiary hospitals located in Beijing. Among them, the urology department of the First Medical Center has 140 beds and the Third Medical center has 145 beds. The Third Affiliated Hospital of Naval Military Medical University is also a tertiary hospital located in Shanghai. The urology department has 47 beds. The interviews were first conducted at the First Medical Center of PLA General Hospital. After information saturation was achieved, the interviews were conducted at the Third Medical Center of PLA General Hospital and the Third Affiliated Hospital of Naval Military Medical University to expand the diversity of the samples.

### Setting

ZYA conducted face-to-face interviews at PLA General Hospital and QWZ conducted those at the Third Affiliated Hospital of Naval Military Medical University. Both these researchers were male, with a MD degree in urology, had extensive clinical experience and systematic understanding and training of the qualitative study. They were not directly involved in the treatment of the patients and keep objective during the interview. Before the formal interview began, the interviewers introduced themselves to gain the trust of the patients and establish a good relationship. They would also introduce the purpose of the study and obtained written informed consent from the participants. All interviews were conducted in a quiet office in the ward to avoid any interruptions. All participants completed the study and none dropped out. Interviews lasted an average of 21.5 ± 8.4 min (range 11–63 min).

### Data analysis

The investigators transcribed recordings into text in Word after the interview. Inductive thematic analysis was performed to analyze the data using the NVivo V.12 software (QSR International, Melbourne, Australia). Three male researchers (ZYA, QWZ, and JPS) formed an analysis team and analyzed these materials separately. The analysis steps included preliminary classification and coding, extracting themes, reviewing potential themes, naming the themes, and generating the final report. After the first interview, the content was reviewed and coded by the analysis team to determine the preliminary coding framework. Thereafter, interviews, transcription of the recordings, and coding were simultaneously conducted. Group discussion was conducted for every five cases to review the coding and to achieve consistency. Themes were continuously reviewed until no new theme emerged; at this point, theme saturation was achieved. The final thematic report was agreed upon by all researchers via group discussions.

## Results

Between July 2021 and November 2022, 46 patients with BPH underwent semi-structured interviews at the three tertiary hospitals. Of them, 36 were recruited at the First Medical Center of PLA General Hospital and five patients each were recruited at the Third Medical Center of PLA General Hospital and the Third Affiliated Hospital of Naval Military Medical University. Table 1 presents the demographic characteristics of the patients. Table 2 presents the clinical data of the patients. Table 3 categorizes the themes, sub-themes, and sample quotations of the life and treatment experiences of the patients.

**Table 1:** Patients’ demographic characteristics

Characteristics	N(%)
Age distribution(years)	
≤ 59	7(15.2)
60–69	27(58.7)
70–79	11(23.9)
≥ 80	1(2.2)
Education level	
Elementary school	6(13.0)
Junior school	8(17.4)
Senior high school	13(28.3)
University and above	19(41.3)
Marital status	
Married	45(97.8)
Unmarried or single	1(2.2)
Work status	
Retirement	40(87.0)
Unretirement	5(10.9)
Jobless	1(2.2)
Type of treatment	
Local patients	14(30.4)
Ecdemic patients	32(69.6)
Type of medical insurance	
Medical insurance for urban workers	30(65.2)
Medical insurance for urban residents	2(4.4)
New rural cooperative medical insurance	7(15.2)
Free medical service	6(13.0)
Self-paying	1(2.2)

Ecdemic patients: Patients who were forced to travel to big large cities to seek better treatment

Medical insurance for urban workers: Mainly for employees of urban employers, urban residents of the working age, and retirees who enjoy pensions

Medical insurance for urban residents: For people who are not covered by the medical insurance for urban workers, such as rural residents, urban non-working residents, school students, preschool children, etc

New rural cooperative medical insurance: A type of health insurance advocated, organized and sponsored by government, with rural residents’ voluntary enrolment

Free medical service: A social security system in which the state provides free medical services to state workers through the medical and health departments

**Table 2:** Patients’ clinical data

Variables	N(%)
Disease duration(years)	
1–5	20 (43.5)
6–10	16 (34.8)
11–15	5 (10.9)
16–20	4 (8.7)
> 20	1 (2.2)
IPSS(points)	
8–19 (Moderate)	8 (17.4)
20–35 (Severe)	38 (82.6)
QOL(points)	
3 (Mixed)	5 (10.9)
4 (Mostly dissatisfied)	49 (8.7)
5 (Unhappy)	24 (52.2)
6 (Terrible)	13 (28.3)
Comorbidities	
Acute urinary retention	17 (37.0)
Hydronephrosis	3 (6.5)
Urinary calculi	6 (13.0)
Urinary infection	7 (15.2)
Hematuria	4 (8.7)
No comorbidities	9 (19.6)

IPSS (International Prostate Symptom Score): 0–7, mildly symptomatic; 8–19, moderately symptomatic; 20–35, severely symptomatic

QOL (Quality of Life): 0, delighted; 1, Pleased; 2, Mostly satisfied; 3, Mixed; 4, Mostly dissatisfied; 5, Unhappy; 6, Terrible

**Table 3:** Themes, subthemes and sample quotations of patients’ life and treatment experience

Themes	Sub-themes	Sample quotations
Disturbed life	1. Poor sleep quality	“I cannot sleep well. How can I sleep? I peed 20 or 30 times a night. I used a bottle to get urine at night, but only two drops at a time. No sooner had I put the jar down then I had to pee again, not even for five minutes” – Patient 15 “She has a bed and I have a bed. She didn’t sleep very well. And when I woke up at night, it affected her even more.” – Patient 6
	2. Decreased sexual function	“I haven’t had sex for more than a year, and I couldn’t even get an erection in the last two or three months” – Patient 17 “The sex life is long gone, and the sexual function is no longer good. The prostate must affect this” – Patient 33
	3. Travel inconvenience	“I didn’t take the bus when I came to Beijing, because the bus seldom stopped on the road. If I have no place to pee, I can’t hold on. This is the obvious trouble” – Patient 6 “I had no choice. There was no bathroom on the street, so I had to pee in the woods by the road” – Patient 8 “Once, I was driving outside and got stuck in traffic and had nowhere to go. I held in my urine for a long time and ended up with acute urinary retention” – Patient 34
	4. Limited social life	“I’m afraid to go too far, and once I get out, I have to keep an eye on where the bathroom is” – Patient 4 “Sometimes I couldn’t find the toilet, so I had to pee in my pants. It was very embarrassing. So I don’t want to be with others” – Patient 21
	5. Affecting work	“I had to ask the students for a leave because I couldn’t hold my urine in class. I said I had to go out for a while” – Patient 4 “The meeting lasted a long time……I had to go to the bathroom every hour, so I didn’t know what the meeting was about” – Patient 18
Mental burden	1. Long term negative emotions	“I always have a feeling of depression, but cannot say anything out. I can only console myself with the fact that I am old and just have a small problem” – Patient 1 “I just want to have surgery. I couldn’t stand it. That feeling is really worse than death” – Patient 15
	2. Occasional anxiety	“A bad night’s sleep affects my mood. But when the daytime came, it seemed to be forgotten” – Patient 4 “Only when I couldn’t pee, I feel bad and irritable” – Patient 13
Disease cognition and communication	1. Lack of knowledge of the disease	“I just know it is prostatic hyperplasia, but I don’t understand its mechanism or its harm” – Patient 5 “I don’t know. I heard from my son-in-law that hyperplasia was a tumour” – Patient 20 “I know nothing about this disease. I don’t understand this” – Patient 28
	2. Lack of knowledge of the surgery	“No, I’m completely blank about the surgery, and I hadn’t even planned to be hospitalized” – Patient 11 “I think it is a total resection of the prostate. There is no substitute for parts of the human body and surgery can affect life span” – Patient 36
	3. Lack of effective communication	“As this is a private matter, I do not like to talk about it and I do not like to share my negative aspects with others” – Patient 8 “I didn’t tell my family until I couldn’t pee, and then my daughter took me in for a check-up” – Patient 20 “I have not communicated with my children about these things and I think it is not a big disease. If I can bear it, I’ll bear it” – Patient 3
	4. Difficulty in obtaining information	“Everyone has this problem, but some people say it is prostatitis and can be treated by sitz bath” – Patient 4 “I have read some introductions on the Internet. But some things on the Internet are one-sided” – Patient 30
Delayed treatment	1. Insufficient attention	“I lacked this awareness (of seeking treatment) and thought my health was not bad” – Patient 3 “I didn’t care. I didn’t take it seriously” – Patient 42
	2. Concerns about surgery	“I’m worried about that it would relapse soon after the surgery, or that it would be incontinence” – Patient 27
	3. Poor economic or medical conditions	“I didn’t go to see a doctor in my hometown. I just put up with it because I didn’t have money” – Patient 28
Medication status	1. Discontinuation medication	“There was no difference in subjective feelings between taking and not taking pills. Then I stopped taking them” – Patient 34 “I don’t usually take them (tamsulosin) because they make me dizzy. Sometimes blood pressure drops after taking these pills” – Patient 30
	2. Irregular medication	“I just take some medicine when it is painful to urinate. No pain, no medicine. I don’t take my medication consistently” – Patient 22
	3. Sub-standard medication	“I went to the drugstore and bought some medicine by myself. But I didn’t go to the hospital or see a doctor. I bought it by myself” – Patient 13
	4. Never taken medicine	“I asked the doctor whether the medicine would make my prostate smaller. He said no, so I did not take it” – Patient 36
Hospital visits barriers	1. Severe dependence on children	“My daughter helped me register and queue up. I couldn’t do it. I couldn’t even take the subway here by myself. I couldn’t find it” – Patient 24 “When I couldn’t pee, I called my son and waited to see a doctor. He had something to do at work and didn’t take me to hospital until 2 p.m” – Patient 15 “My visit to see a doctor wasted my children’s time and had a great impact on their work and rest” – Patient 2
	2. Lack of outpatient guidance	“The hospital is too big and you can’t find the examination room without guidance. So you really need someone to guide” – Patient 33 “Sometimes I didn’t know where to go for formalities and queues, and I couldn’t find the right place” – Patient 4 “The barrier is to register online or get a number on the queueing machine. Some procedures are really difficult for older people” – Patient 5
	3. Long waiting time	“There were so many people here that it took days to get in line” – Patient 3 “I came here in August and the doctor arranged an ultrasound and a urodynamic. But I could not do urodynamic until September 7th” – Patient 32
Medical insurance issues	1. Low reimbursement ratio for ecdemic medical insurance	“This belongs to ecdemic medical insurance. I can get 30% reimbursement when I go back” – Patient 1 “I can get reimbursement for my ecdemic medical insurance here, but only a little money. What’s the use of that?” – Patient 28
	2. Complicated medical insurance formalities	“The local medical insurance system was always in a state of maintenance and I failed to apply for ecdemic medical insurance when I came here” – Patient 25 “I come from my hometown and I need a temporary residential permit for reimbursement. But I don’t have a temporary residential permit now and I’m also not clear about the formalities” – Patient 32
	3. Requirement of a referral certificate	“With a referral certificate, I can get 20% more reimbursement in our local” – Patient 11 “It is not easy to get a referral for this disease, because this disease (BPH) is not a serious disease in our hometown” – Patient 33

### Disturbed life

#### Poor sleeping quality

In this study, 36 patients (78.3%) reported nocturnal urination with a frequency of three to twenty times. Further, fifteen patients (32.6%) mentioned that their wives’ sleep was disturbed because of their frequent nocturnal urination; to avoid this situation, four of these patients chose to sleep separately from their wives.

#### Decreased sexual function

Except for two patients (4.4%) who had a normal sexual life, all patients experienced decreased sexual function and sexual frequency, including shortened time, ejaculatory pain, and incomplete erection.

#### Traffic inconvenience

Patients mentioned that they were often unable to go to the toilet in time when traveling long distances by car and particularly by bus, making them suffer a lot. Therefore, they were reluctant to travel and extremely careful about their transportation choice. In our study, two patients (4.4%) experienced acute urinary retention because of traffic jams while riding a vehicle and three patients (6.5%) used unconventional urination methods inside the car or by the side of the road.

#### Limited social life

Thirty patients (65.2%) mentioned that they tended to avoid going out and had reduced social activities because they felt that frequent toilet visits could damage their personal image when participating in some activities. Further, they feared that they might have urgent urinary incontinence when a toilet cannot be found in time, resulting in an embarrassing situation. In addition, they were worried that the smell of urine on their pants could affect others.

#### Affecting work

Thirteen patients (28.3%) developed severe LUTS before their retirement, affecting their work because they were often interrupted by bouts of frequent urination and dysuria during meetings, classes, or other work activities.

### Mental burden

#### Long-term negative emotions

Thirteen patients (28.2%) had long-term negative emotions such as mental depression and anxiety owing to disturbance of LUTS. None of these patients had received professional psychological counselling and their psychological problems had been ignored for a long time.

#### Occasional anxiety

Ten patients (21.7%) experienced temporary impatience and annoyance when they were troubled by LUTS. However, they did not believe that their mental state was abnormal because their negative feelings were often automatically relieved as the symptoms disappeared.

### Disease cognition and communication

#### Lack of knowledge of the disease

Most patients (69.6%) had a superficial understanding of BPH and believed that these micturition symptoms were a natural phenomenon of aging and did not pay much attention to them. Ten patients (21.7%) were not even aware of what disease they had and had no idea about BPH. Further, three patients (6.5%) had a misconception about BPH because they associated it with tumours.

#### Lack of knowledge of the surgery

Approximately 50% of the patients (47.8%) lacked basic knowledge of the surgical treatment options for BPH even after hospitalization. Only 20 patients (43.5%) had a further understanding of the surgery and were aware that they would undergo minimally invasive surgery. Four patients had some wrong views, resulting in resistance to the operation.

#### Lack of effective communication

Seventeen patients (40.0%) believed that talking about these private symptoms would hurt male self-esteem and were reluctant to discuss this awkward topic with others. Thirteen patients (28.3%) chose to hide their condition and not seek their children’s help owing to the privacy of the symptoms and male self-esteem; this delayed disease diagnosis and treatment. The other patients normally did not take the initiative to discuss the illness with their children until the they had an urgent need for medical treatment.

#### Lack of effective communication

Patients did not have access to channels to obtain effective information about the disease. Although doctors were the primary information source for patients with BPH, only a few patients gained a clear understanding of their disease during the fast-paced hospital visit. Some patients would also consult their friends about the treatment options for BPH; however, they would receive incorrect information. In this study, sixteen patients (34.8%) obtained information via television or the Internet; five patients (10.9%) did not believe the information because of advertising and false publicity. Only one patient had attended popular health sciences lectures.

### Delayed treatment

In this study, three main reasons were identified for delayed doctor visits: insufficient attention, concerns about surgery, and poor economic or medical conditions. First, patients did not pay enough attention to this condition even though it had a serious impact on their QOL. They believed that LUTS was a normal aging phenomenon in elderly men; further, their awareness of seeking treatment was weak. Second, some patients were afraid to receive surgery owing to fear of surgical complications and adverse effects, including postoperative recurrence, urinary incontinence, and sexual dysfunction. Finally, poor family economic or lack of medical resources also resulted in a few patients not seeking timely medical treatment.

### Medication status

#### Discontinuation of medication

The medication prescribed for most patients in this study was unsatisfactory. Nineteen patients (41.3%) stopped taking drugs one month before hospitalization owing to poor effects, inefficacy, or obvious side effects. These drugs included Chinese patent medicine, α-receptor blockers, and 5α-reductase inhibitors.

#### Irregular medication

Six patients (13.0%) had good efficacy; however, their symptoms relapsed or worsened owing to irregular medication. Only thirteen patients (28.3%) regularly took the medicine as prescribed by the doctor, and four mentioned that the medicine was no longer effective.

#### Sub-standard medication

Four patients (8.7%) did not receive formal treatment at the initial stages of the disease; however, they purchased drugs based on their own experience, which may have covered up the disease condition.

#### Never taken medicine

Eight patients (17.4%) had never taken any medication owing to insufficient attention or because they were worried about side effects.

### Hospital visits barriers

#### Severe dependence on children

In China, elderly individuals require children’s help during hospital visits. Owing to a decline in mobility and cognitive ability, most patients (74.0%) were unable to independently finish the medical treatment process and needed their children’s help in choosing hospitals, medical registration, transportation, outpatient navigation, and so on. In particular, unfamiliar cities and hospitals made ecdemic patients more dependent on their children.

#### Lack of outpatient guidance

Because most patients were unfamiliar with the hospital environment and procedures, they spent a lot of their energy looking for consulting rooms, examination rooms, and other windows and were confused about the cumbersome procedures. Further, some elderly patients could not enjoy the convenience of intelligent hospitals because they were unaware of the use of smartphones and intelligent machines.

#### Long waiting time

Many examinations required the patients to wait for several days after an appointment; as a result, they had to visit the hospital many times, such as for urodynamics studies and MRI. Further, patients often had to wait many days to be admitted to a ward after making an appointment for hospitalization. These barriers increased financial burden and caused trouble to the patients, particularly to ecdemic patients.

### Medical insurance issues

#### Low reimbursement ratio for ecdemic medical insurance

Only 50% of the patients were aware of the reimbursement rates of Medicare, which ranges from 30 to 95%. The Medicare reimbursement rates of ecdemic patients were lower than those of local patients owing to the hierarchical medical policy.

#### Complicated medical insurance formalities

Each patient encountered different problems when applying for medical insurance owing to policy and regional differences. In some areas, the Internet could be used to handle medical insurance procedures; however, in some other areas, the medical insurance Internet system was always unavailable. Ecdemic patients needed some additional supporting materials to apply for medical insurance, including a temporary resident permit or a referral certificate.

#### Requirement of a referral certificate

Ecdemic patients would bear a higher self-payment ratio if they failed to provide a referral certificate. However, referral certificates are not easily available for patients with BPH because BPH is not considered a serious disease that requires transferring to a tertiary hospital for treatment. As a result, patients were troubled by this issue when they visited other hospitals for medical treatment.

## Discussion

Our study was the first study to use the qualitative research method to investigate the life troubles and medical treatment barriers of hospitalized patients with BPH in China. Through inductive analysis, we established seven themes: disturbed life, mental burden, disease cognition and communication, delayed treatment, medication status, hospital visits barriers, and medical insurance issues.

In this study, the severity of nocturia in some patients is beyond our expectations. Frequent nocturia, accompanied by incomplete emptying, dysuria, and odynuria, seriously hampered the sleep quality of patients. Further, getting up frequently during the night reduced the sleep quality of the wife, possibly leading to relationship strain between the couple [14–17]. Most patients in our study were also potentially threatened by nocturia. Because frequently getting up at night is an important risk factor for falls among elderly patients, increasing the risk of fractures and death [18, 19].

The effect of LUTS on overall life is also reflected in patients’ severe travel restrictions and qualms about transport, which have been rarely reported before. In our interviews, two patients suffered from acute urinary retention owing to traffic jams, and three patients were forced to adopt unconventional urination methods, including using a water bottle in the car or urinating by the side of the road. These situations greatly affect the dignity of patients and will force them to travel more cautiously. When going out for a short distance, the first task was to determine the location of the toilet to avoid acute urinary retention or incontinence. Further, patients reduced their social activities as much as possible to avoid embarrassing situations because they were worried about not being able to find a toilet and the frequent urination symptoms. These problems may result in potential obstacles for them to go out to visit a doctor. A study also found that the need to plan and to find toilets and the lifestyle changes that accompany LUTS were also seen as both a consequence of LUTS and a cause of distress. For some men, LUTS had led to anxiety and embarrassment about social situations and had stopped them from going on holiday [20].

For mental burden, the persistent suffering of symptoms makes some patients feel negative emotions, mainly manifested as anxiety, depression, and annoyance. However, most patients bear this mental burden silently because of the backwardness of the team construction of Chinese psychologists and neglection of the mental health of patients. A follow-up survey in Taiwan revealed that patients with BPH have a 1.87 times higher risk of being diagnosed with depression than those without BPH [21]. Therefore, in addition to paying attention to LUTS, clinicians should pay better attention to the mental health of patients.

Although the importance of health education and lifestyle advice is emphasized in the guidelines, patients generally do not receive adequate and effective health education and even have misconceptions about disease and surgery. The primary reason for this may be that Chinese doctors spend less time on doctor–patient interaction and disease explanation. Further, patients find it difficult to understand the professional interpretations and medical terms of doctors [22]. Owing to the lack of effective methods to popularize health sciences for the public in medical units and society, patients do not have sufficient access to reliable medical information. Current TV stations lack high-quality and popular medical sciences programs; further, some local TV stations broadcast misleading commercial medical programs and advertisements. Moreover, some patients may obtain inaccurate disease information and misleading commercial advertisements by searching through the Internet. Therefore, uncensored media information negatively interferes with the treatment decisions of patients [23, 24].

We found that the patient’s treatment regimen was dominated by medical therapy, but the overall treatment effect was unsatisfied. Moreover, the lack of health education leads to weak awareness of health management and poor compliance with treatment regimens. For example, irregular medication can lead to recurrent symptoms and decreased medicine effects. As the efficacy of the drug is no longer significant, patients gradually lose confidence in treatment and eventually choose to stop taking medicine. In addition, some patients also buy medications based on their own experience, while others insist on not taking medicine. These all occur over a longer period of time, but BPH itself presents with private symptoms, and the embarrassment of discussing LUTS or sex prevents patients from communicating with others and seeking medical help [8]. As these symptoms are perceived as private, there is a lack of effective supervision of patients’ disease management, failing to finish the treatment.

The majority of patients enrolled in our study had previously visited a doctor (88.9%), which is higher than the low rates reported in other studies [5, 25, 26]. One reason is that other studies have focused on patients in the community. The reason for the low rate of hospital visits among patients in the community is that they have milder symptoms and lack motivation to seek medical treatment. However, our research group was hospitalized patients who chose surgical treatment. This group itself has a longer medical history and more severe symptoms. Most of these patients had visited the hospital before finally choosing surgery, either to seek other treatments that proved ineffective or lost confidence, or to consult about problems related to surgical treatment. These are the reasons for the higher rate of previous hospital visits among patients in this study. However, the patients had two kinds of delayed treatment in the illness process. In the early disease stage, patients’ ignorance or misunderstanding of the disease as well as lack of attention could have led to delays in the first hospital visit and drug treatment. In the later stage of the disease, patients became extremely hesitant about undergoing surgery because of their ignorance of the surgery and worries about the associated complications, which eventually led to delayed surgical treatment. Further, the limitations of economic conditions and the local medical level also affected the early diagnosis and treatment of patients.

One of the most prominent obstacles faced by elderly Chinese patients during hospital visits is their dependence on children. Owing to reduced ability to perform activities and unfamiliarity with network technology, elderly patients face difficulties in finding proper hospitals, registration, transportation, navigation, and other aspects. The support of family and children can considerably reduce the difficulty faced in seeking medical treatment. However, this dependence also causes some problems, such as children being unable to take patients to the hospital on time because of their busy work schedules. In addition, children needed to ask for frequent leaves, resulting in them sacrificing their work and rest time and reducing their income. We also found that some children of empty nesters were less aware of their father’s health because they have been working in other places for a long time. These patients also need some social help because their children are often unable to give them timely care and help.

We also identified that the problems of ecdemic medical care were prominent, including low reimbursement ratio, the requirement of a referral certificate, and complicated formalities for applying for medical insurance. China is promoting the implementation of the hierarchical medical system and medical insurance reform to solve the social difficulties of ecdemic medical care. In particular, the main reasons for differences in reimbursement ratio and the requirement of referral certificates in different levels of hospitals are to promote completion of disease treatment in a primary hospital, avoid patients swarming into large hospitals, and realize the rational allocation of medical resources. However, the current contradiction is between patients’ demand for high-level medical treatment and the weak strength of primary hospitals. Therefore, this contradiction warrants reformation of the whole medical system and social progress.

The seven themes summarized in the present study reflect the current difficulties faced by Chinese middle-aged and elderly patients with BPH. These difficulties are related to individuals and families, hospitals and society, and social development and institutional reform. Based on the abovementioned difficulties, we proposed six feasible solutions.

First, strengthen the science popularization of BPH. The objects of science popularization are mainly patients and their families; therefore, the publicity methods should be extremely easy to understand. For the elderly, community lectures can be conducted, brochures can be handed out, and medical TV channels and programs can be broadcasted. For young and middle-aged people, internet publicity can be strengthened to improve people’s care for their elders and increase awareness of disease prevention. Meanwhile, it is also important to reduce the influence of advertisements and commercial interests.

Second, strengthen the psychological counselling of patients. Owing to the lack of professional psychological doctors in China, doctors at all levels should strengthen communication with patients to relieve their psychological anxiety, help them establish a correct view of the disease, and encourage them to actively seek medical treatment.

Third, increase investments in basic health units. At present, there are 3,275 tertiary hospitals in China, accounting for 0.32% of hospitals; 33,295 primary and secondary hospitals, accounting for 3.2% of hospitals; and 977,790 primary health institutions, accounting for 94.8% of hospitals [27]. Although there are a large number of basic medical units, these units have not completely played their roles and patients still prefer tertiary hospitals. Primary hospitals and health institutions should receive more medical resources so that they can provide correct treatment and health guidance to patients in the early disease stages and build a rational hierarchical medical system.

Fourth, improve the family doctor system. As a link between patients and hospitals, family doctors can provide patients with active medical services, resulting in early detection and treatment of diseases and strengthening the health management and follow-up of patients. Simultaneously, family doctors can connect with secondary and higher-level hospitals to provide green referral channels for patients.

Fifth, the outpatient department of hospitals should provide more intimate medical help. At present, many elderly patients are unfamiliar with the hospital environment and treatment process. To solve this issue, more medical service guides and information desks are warranted.

Finally, strengthen social support and care for the elderly. China is now an aging society, and the proportion of empty nesters has drastically increased. Social welfare organizations should create professional nursing teams so that patients can get professional accompany and care when seeking medical treatment or surgery, thereby relieving the pressure on children to support their parents.

To our knowledge, this study is the first qualitative interview-based investigation of the LUTS symptoms and medical challenges experienced by hospitalized patients with BPH in China. By gathering information directly from patients, we were able to develop a professional research report with significant practical implications for improving patient treatment. This patient-centered approach is a unique innovation and advantage of our study, as previous research has primarily focused on the perspectives of medical professionals. Overall, our study sheds light on the experiences of patients with BPH and provides valuable insights for improving their care.

## Strengths and limitations

The present study also has limitations. The survey was conducted only in large cities, and some variations may arise due to differences between urban and rural regions. However, the 46 patients in this study were selected from 39 cities in 20 provinces, which provides a representative sample and helps to reduce these variations to some extent.

## Conclusions

In this study, we identified seven main aspects of problems and obstacles faced by patients with BPH. Based on these findings, we proposed feasible suggestions, including strengthening the science popularization of BPH and psychological counselling of patients, increasing investments in basic health units, improving the family doctor system and hospital outpatient service, strengthening social support and care for the elderly. These suggestions aim to optimize the medical system, promote medical reform and social progress, and improve the difficulties faced by patients with BPH. Our study provides a reference for future research to comprehensively explore the challenges encountered by patients with BPH in China.

### Electronic supplementary material

Below is the link to the electronic supplementary material.

Supplementary Material 1

Supplementary Material 2

## Data Availability

Data can be obtained from corresponding author upon reasonable request.

## Abbreviations

BPH: benign prostatic hyperplasia
LUTS: lower urinary tract symptoms
IPSS: international prostate symptom score
QOL: quality of life
