# Supplementary material for: Life disturbance and hospital visit experiences among Chinese patients with benign prostatic hyperplasia: a qualitative study
Source: BMC Prim Care. 2024 May 3;25:149. doi: 10.1186/s12875-024-02378-5 (PMC11069269; doi:10.1186/s12875-024-02378-5)
Supplement: Supplementary file 1 — Supplementary Material 1 [file 12875_2024_2378_MOESM1_ESM.docx]

**Interview guide**

- When did you first start experiencing symptoms like frequent urination, urgent urination, dysuria, and so on?
- Which symptom do you find the most bothersome?
- In what ways have these symptoms affected your daily life?
- Have these symptoms had an impact on your mood or spirit?
- Have you discussed this illness with your friends or family?
- What do you know about surgery for benign prostatic hyperplasia?
- Do you have any expectations or concerns about the surgery for benign prostatic hyperplasia?
- Have you taken any initiative to learn more about it? If so, how did you obtain this information?
- Have you paid attention to these symptoms over the years?
- Have you sought medical attention in a timely manner? If not, what has prevented you from doing so?
- Have you taken any medications in the last month? What was the effect?
- Which hospitals have you visited?
- Why did you choose to have surgery at this hospital?
- What challenges or difficulties have you experienced in seeking medical care?
- Are you satisfied with the communication you have had with your doctor?
- What type of medical insurance do you have?
- What is the reimbursement rate of your medical insurance?
- Have you experienced any problems with the medical insurance process?
- Dose the cost of medical treatment have a significant impact on your life?
