# Supplementary material for: Life disturbance and hospital visit experiences among Chinese patients with benign prostatic hyperplasia: a qualitative study
Source: BMC Prim Care. 2024 May 3;25:149. doi: 10.1186/s12875-024-02378-5 (PMC11069269; doi:10.1186/s12875-024-02378-5)
Supplement: Supplementary file 2 — Supplementary Material 2 [file 12875_2024_2378_MOESM2_ESM.docx]

**International Prostate Symptom Score**

| Have you had any of the following symptoms in the past 1 month? | Not at all | Less than 1 in 5 times | Less than half the time | About half the time | More than half the time | Almost always | Symptom score |
| --- | --- | --- | --- | --- | --- | --- | --- |
| 1. How often have you had the sensation of not emptying your bladder? | 0 | 1 | 2 | 3 | 4 | 5 |  |
| 1. How often have you had to urinate less than every two hours? | 0 | 1 | 2 | 3 | 4 | 5 |  |
| 1. How often have you found you stopped and started again several times when you urinated? | 0 | 1 | 2 | 3 | 4 | 5 |  |
| 1. How often have you found it difficult to postpone urination? | 0 | 1 | 2 | 3 | 4 | 5 |  |
| 1. How often have you had a weak urinary stream? | 0 | 1 | 2 | 3 | 4 | 5 |  |
| 1. Do you often have to force yourself to start urinating? | 0 | 1 | 2 | 3 | 4 | 5 |  |
| 1. How many times do you typically get up at night to urinate? | None | 1 time | 2 times | 3 times | 4 times | 5 times or more |  |
|  | 0 | 1 | 2 | 3 | 4 | 5 |  |
| Total IPSS score = | | | | | | | |

Score: Mild, 0-7; Moderate, 8-19; Severe, 20-35

**Quality of Life Score**

| Quality of life due to urinary symptoms | Delighted | Pleased | Mostly satisfied | Mixed | Mostly dissatisfied | Unhappy | Terrible |
| --- | --- | --- | --- | --- | --- | --- | --- |
| If you were to spend the rest of your life with your urinary condition just the way it is now, how would you feel about that? | 0 | 1 | 2 | 3 | 4 | 5 | 6 |
| QOL score = | | | | | | | |
